# Female rats with severe left ventricle volume overload exhibit more cardiac hypertrophy but fewer myocardial transcriptional changes than males

**DOI:** 10.1038/s41598-017-00855-9

**Published:** 2017-04-07

**Authors:** Catherine Beaumont, Élisabeth Walsh-Wilkinson, Marie-Claude Drolet, Élise Roussel, Marie Arsenault, Jacques Couet

**Affiliations:** Groupe de recherche sur les valvulopathies, Centre de Recherche, Institut universitaire de cardiologie et de pneumologie de Québec, Université Laval, Quebec City, Canada

## Abstract

Aortic valve regurgitation (AR) imposes a volume overload (VO) to the left ventricle (LV). Male rats with a pathological heart overload usually progress more quickly towards heart failure than females. We examined whether a sexual dimorphism exists in the myocardial transcriptional adaptations to AR. Adult Wistar male and female rats either underwent a sham operation or were induced with AR and then followed for 26 weeks. Female AR rats gained relatively more LV mass than males (75 vs. 42%). They had a similar increase in LV chamber dimensions compared to males but more wall thickening. On the other hand, fatty acid oxidation (FAO)-related LV enzyme activity was only decreased in AR males. The expression of genes encoding FAO-related enzymes was only reduced in AR males and not in females. A similar situation was observed for the expression of genes involved in mitochondrial biogenesis or function as well as for genes encoding for transcription factors implicated in the control of bioenergetics and mitochondrial function (Errα, Errγ or Pgc1α). Although females develop more LV hypertrophy from severe VO, their myocardial gene expression remains closer to normal. This could provide survival benefits for females with severe VO.

## Introduction

Cardiac hypertrophy (H) is used as a prognostic indicator of progression towards heart failure (HF). HF occurs in women as frequently as in men but later in life and less often from ischaemic causes^1^. Women are more likely than men to develop HF with preserved ejection fraction (HFpEF) associated with diastolic dysfunction and concentric left ventricle (LV) remodelling. Unfortunately, no treatment has been proven effective for HFpEF. LVH becomes more prevalent in women after menopause and constitutes the strongest mortality predictor from HF^2^.In pressure overload (PO) diseases such as aortic valve stenosis or arterial hypertension, female patients usually develop more LVH, although they have better EF and less myocardial fibrosis than male patients^3, 4^. Hypertensive women also develop more LVH compared to men even for similar blood pressure levels^4^. Volume overload (VO) typically induces eccentric LV hypertrophy. Sex differences in patients with volume overload (VO) diseases (heart valve regurgitations) remain mostly unstudied.

In pre-clinical heart disease models in rodents (either rat or mouse), oestrogens have been shown to slow the development of LVH, delay HF and improve survival^5, 6^. For instance, male mice with transverse aortic constriction (TAC; a PO model) develop concentric LVH sooner than females^7^. LV remodelling in these male TAC mice later evolves towards eccentric LVH and HF symptoms^8^. Genes associated with extracellular matrix (ECM) production or with mitochondrial function are also more strongly modulated in males. Similar observations have been made in several other animal models of LVH and/or HF^5, 6^. Two VO models have been studied in rats. Females with arteriovenous fistula (AVF) develop a similar amount or more LVH than males^9–12^. Progression towards HF is slower for females, and their survival rate is higher^13^. In the aortic valve regurgitation (AR) rat model, we observed that females developed more LVH than males and that removing oestrogens by performing an ovariectomy did not influence LVH development^14^.

In both pre-clinical PO and VO rodent models, the development of HF seems to be slower in females, but this may not be directly linked to levels of cardiac hypertrophy. Therefore, we wanted to investigate sex differences in myocardial transcriptional adaptations to severe VO from severe AR. We demonstrate that LV gene expression in rats with severe AR is significantly less altered in females, although they developed significantly more cardiac hypertrophy than males.

## Results

All sham-operated animals were alive at the end of the protocol. Three male AR rats and one female AR rat died suddenly overnight before the end of the six-month follow-up period. No animals developed signs of heart failure, defined as dyspnoea, peripheral oedema or excessive weight gain. Table 1 summarizes the animal characteristics after 6 months. AR animals tended to have lower body weight (p = 0.13) and to be leaner (p = 0.11).

**Table 1:** Animal characteristics at the end of the study.

Parameters	ShM (n = 11/11)	ShF (n = 7/10)	ArM (n = 7/7)	ArF (n = 7/8)
Body weight, g	703 ± 27.9	349 ± 13.5b	668 ± 45.3	353 ± 15.3b
Heart, mg	1604 ± 70.8	882 ± 30.5b	2282 ± 96.6a	1563 ± 101.1a, b
Ind. Heart, mg/g	2.3 ± 0.11	2.5 ± 0.09	3.4 ± 0.14a	4.4 ± 0.12a, b
LV, mg	985 ± 26.6	591 ± 20.9b	1550 ± 70.1a	1094 ± 66.0a, b
Ind. LV, mg/g	1.4 ± 0.06	1.7 ± 0.06	2.3 ± 0.07a	3.1 ± 0.23a, b
Right ventricle, mg	333 ± 14.0	185 ± 6.7b	424 ± 22.2a	320 ± 40.4a, b
Ind. RV, mg/g	0.48 ± 0.025	0.53 ± 0.022	0.66 ± 0.063a	0.90 ± 0.133a, b
Retroperitoneal fat, g	8.9 ± 1.0	2.8 ± 0.4b	6.7 ± 0.8	2.3 ± 0.5b

Sham: sham-operated controls; Ar: aortic regurgitation; M: male rats and F: female rats. LV: left ventricle, RV: right ventricle and Ind. indexed. Values are expressed as the mean ± SEM. The number of animals per group is indicated in parentheses. ^a^p < 0.05 vs. the corresponding sham group and ^b^p < 0.05 vs. the corresponding male group.

### Cardiac hypertrophy

AR caused a significant gain of cardiac mass for both male and female rats (Table 1). This gain of mass was proportionally more important in females than in males (75% vs. 42%, respectively), although the severity of AR was similar (Table 2). The relative left and right ventricle (RV) weights indexed for body weight were also increased more in female AR rats than in males (LV: 82% vs. 64% and RV: 70% vs. 38%, respectively).

**Table 2:** Echocardiography data and hemodynamic values.

Parameters	ShM (n = 11)	ShF (n = 7)	ArM (n = 7)	ArF (n = 7)
EDD, mm	9.3 ± 0.15	7.9 ± 0.08b	12.1 ± 0.52a	10.4 ± 0.2a, b
ESD, mm	4.4 ± 0.09	3.7 ± 0.08b	7.0 ± 0.45a	5.5 ± 0.19a, b
SW, mm	1.5 ± 0.03	1.3 ± 0.02b	1.5 ± 0.03	1.4 ± 0.03a, b
PW, mm	1.5 ± 0.03	1.4 ± 0.03b	1.6 ± 0.02	1.5 ± 0.02a
LV mass (echo), mg	1109 ± 51.2	724 ± 13.0b	1793 ± 148.9a	1246 ± 85.4a, b
Ind. LV mass (echo), mg/g	1.6 ± 0.08	2.1 ± 0.07b	2.7 ± 0.08a	3.7 ± 0.28a, b
RWT, unitless	0.33 ± 0.004	0.34 ± 0.006	0.26 ± 0.012a	0.28 ± 0.004a
mWS, mmHg.cm^2^	189 ± 1.8	169 ± 1.8	278 ± 15.0a	231 ± 5.6a, b
FS, %	53 ± 0.5	54 ± 0.6	42 ± 1.4a	47 ± 1.0a, b
% regurgitation	na	na	79 ± 4.5	79 ± 7.1
LAD, mm	6.4 ± 0.14	4.3 ± 0.12b	7.9 ± 0.37a	5.7 ± 0.17a, b
MWV, mm/s	20.9 ± 0.58	22.3 ± 0.74	18.0 ± 0.91	21.2 ± 0.80
MPI (Tei index)	0.39 ± 0.019	0.43 ± 0.018	0.52 ± 0.030a	0.52 ± 0.050a
HR, bpm	365 ± 11.0	396 ± 9.0	337 ± 12.9a	380 ± 9.3b
SV, µl	202 ± 3.0	160 ± 0.6b	409 ± 31.0a	260 ± 4.1a, b
CO, ml/min	74 ± 2.7	64 ± 3.2b	137 ± 10.3a	98 ± 2.7a, b
dP/dT+	5470 ± 475	6519 ± 465	4881 ± 354	5679 ± 707
dP/dT−	5389 ± 375	6598 ± 603	4526 ± 271	3467 ± 295a
EDP, mmHg	13.6 ± 0.68	11.3 ± 1.48	17.1 ± 1.06a	13.7 ± 1.60b
SAP, mmHg	125 ± 7.0	123 ± 5.0	131 ± 6.6	128 ± 9.6
DAP, mmHg	78 ± 3.0	75 ± 4.2	58 ± 2.9a	56 ± 5.2a
PP, mmHg	47 ± 5.3	48 ± 3.2	72 ± 4.8a	72 ± 5.0a

EDD: end-diastolic diameter, ESD: end-systolic diameter, SW: septal wall thickness, PW: posterior wall, Ind: indexed, RWT: relative wall thickness, mWS: mean LV wall stress, FS: fractional shortening, LAD, left atrial diameter, MWV: LV mid-wall velocity, MPI: myocardial performance index, HR: heart rate, bpm: beats per minute, SV: stroke volume, CO: cardiac output, dP/dt+ and dP/dt−, maximal and minimal derivative of pressure/time, EDP: LV end-diastolic pressure, SAP: systolic arterial pressure, DAP: diastolic arterial pressure and PP: pulse pressure. Na: not applicable. Values are expressed as the mean ± SEM. The number of animals per group is indicated in parentheses. ^a^p < 0.05 vs. the corresponding sham group and ^b^p < 0.05 vs. the corresponding male group. Echocardiography measurements were obtained under inhaled isoflurane anaesthesia.

### Echocardiographic data

As expected, AR caused marked LV dilatation, as illustrated by an increase in the end-diastolic dimensions (Table 2) that was similar for both sexes (~30%). End-systolic diameters were also significantly increased in male (+59%) and female (+43%) AR rats, resulting in a larger decrease in LV fractional shortening (FS) in males (−20%) than in females (−11%; p = 0.0015). Another index of systolic function, the LV mid-wall velocity (WVI), was lower in the male AR group than in the female group (p = 0.041). Septal wall thickness increased in AR females (p = 0.024) but not in males. A similar trend was also observed for the LV posterior wall (p = 0.12). Both AR males and females displayed eccentric LV remodelling, as evidenced by a decrease in relative wall thickness (RWT; (SW + PW)/EDD) compared to sham animals. The LV remodelling tended to be less eccentric in females than in males, however (p = 0.055). The calculated mean LV wall stress^15^ resulting from AR was moderately higher in males than in females compared to sham controls (+47% vs. +37%, respectively). The myocardial performance index or MPI (a composite index reflecting global systolic-diastolic performance) was similar between male and female AR animals.

### Haemodynamic measurements

Heart rates were lower in AR groups (Table 2). Stroke volume doubled in male AR rats (+102%), whereas it only increased by 63% in females. The calculated cardiac output was significantly increased in both AR groups versus the sham groups and again, more in males than in females (85% vs. 53%, respectively). Invasive intra-cardiac measurements yielded similar dP/dt+ values in all 4 groups, although they tended to be lower in AR groups. The same was true for the dP/dt- values that were significantly lower in both AR groups, this being more evident in females. LV end-diastolic pressures (EDP) tended to be higher in male rats, but they were similarly increased in AR male and female rats. Systolic arterial pressures were similar between the four groups. As expected, diastolic arterial pressure was decreased in AR animals (a feature of the disease) but to the same extent in both sexes, leading to increased pulse pressure.

### Evolution of LV remodelling and function during the course of the study

**Figure 1:**
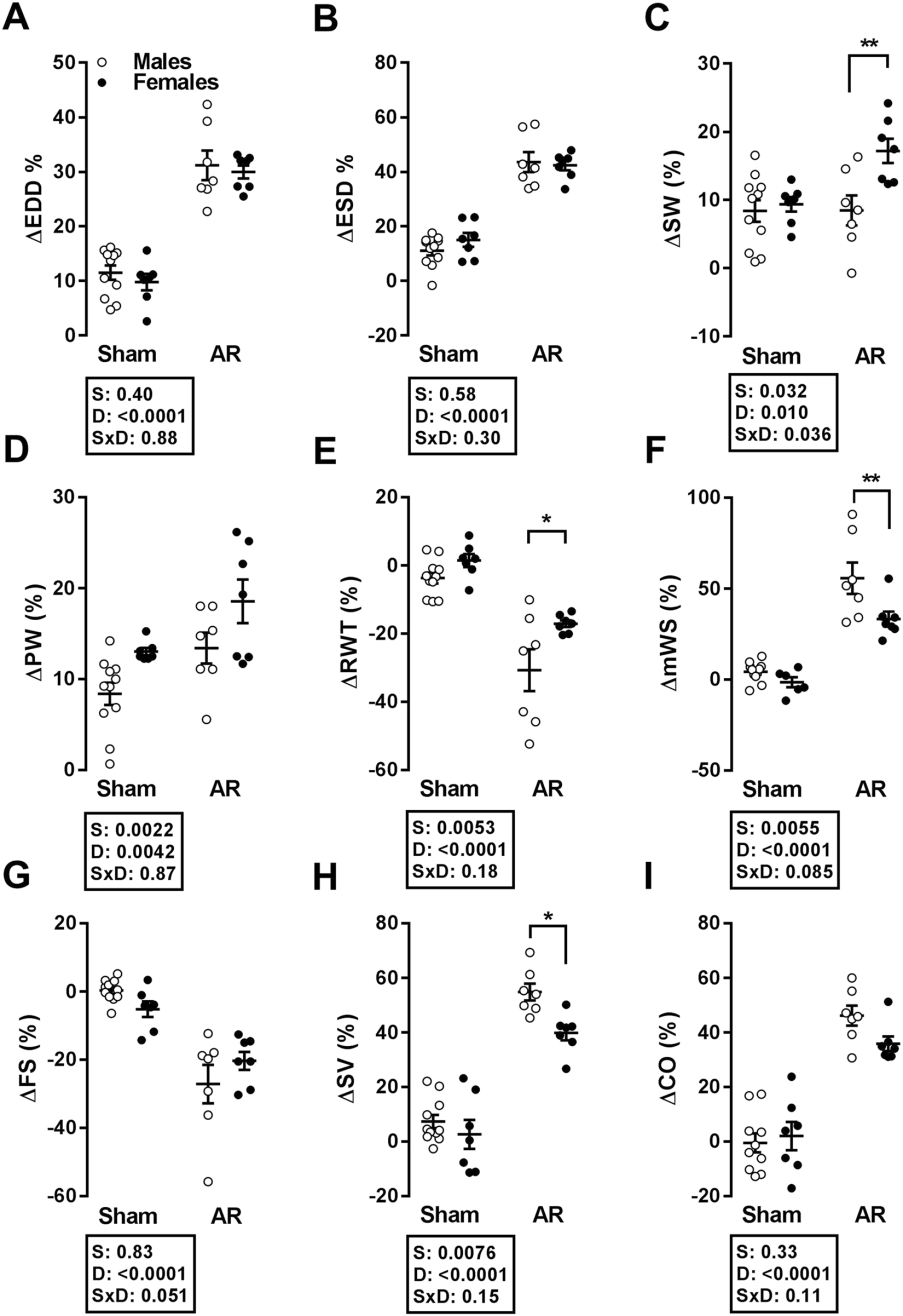
Progression of different LV parameters as evaluated by echocardiography during the duration of the study (26 weeks). Δ: % difference between week 0 and week 26, EDD: end-diastolic diameter (**A**), ESD: end-systolic diameter (**B**), SW: septal wall thickness (**C**), PW: posterior wall thickness (**D**), RWT: relative wall thickness (**E**), mWS: mean wall stress (**F**), FS: fractional shortening (**G**), SV: stroke volume (**H**) and CO: cardiac output (**I**). The results are reported in % as the mean ± SEM (n = 7–11/gr). Probability values from a 2-way ANOVA and Holm-Sidak post-testing to evaluate the p value of sex, (S), disease (D) or disease-sex interactions (S × D) are shown next to each panel.

We measured the variation of different echocardiographic parameters during the 26 weeks of the study by comparing values at the end of the protocol to those at baseline in order to emphasize both the contributions of the volume overload and normal growth on cardiac remodelling. As illustrated in Fig. 1A and B, LV chamber dimensions (systolic and diastolic) increased by the same extent in male and female AR rats. On the other hand, this was accompanied by more thickening of the septum in the ArF group and of the posterior wall in both ShF and ArF groups (Fig. 1C and D). This resulted in less eccentric LV remodelling over time as well as in a smaller increase in stroke volume in AR females compared to males. The increase in LV wall stress was slightly more important in male AR rats than in females (Fig. 1F).

### Tissue analysis

**Figure 2:**
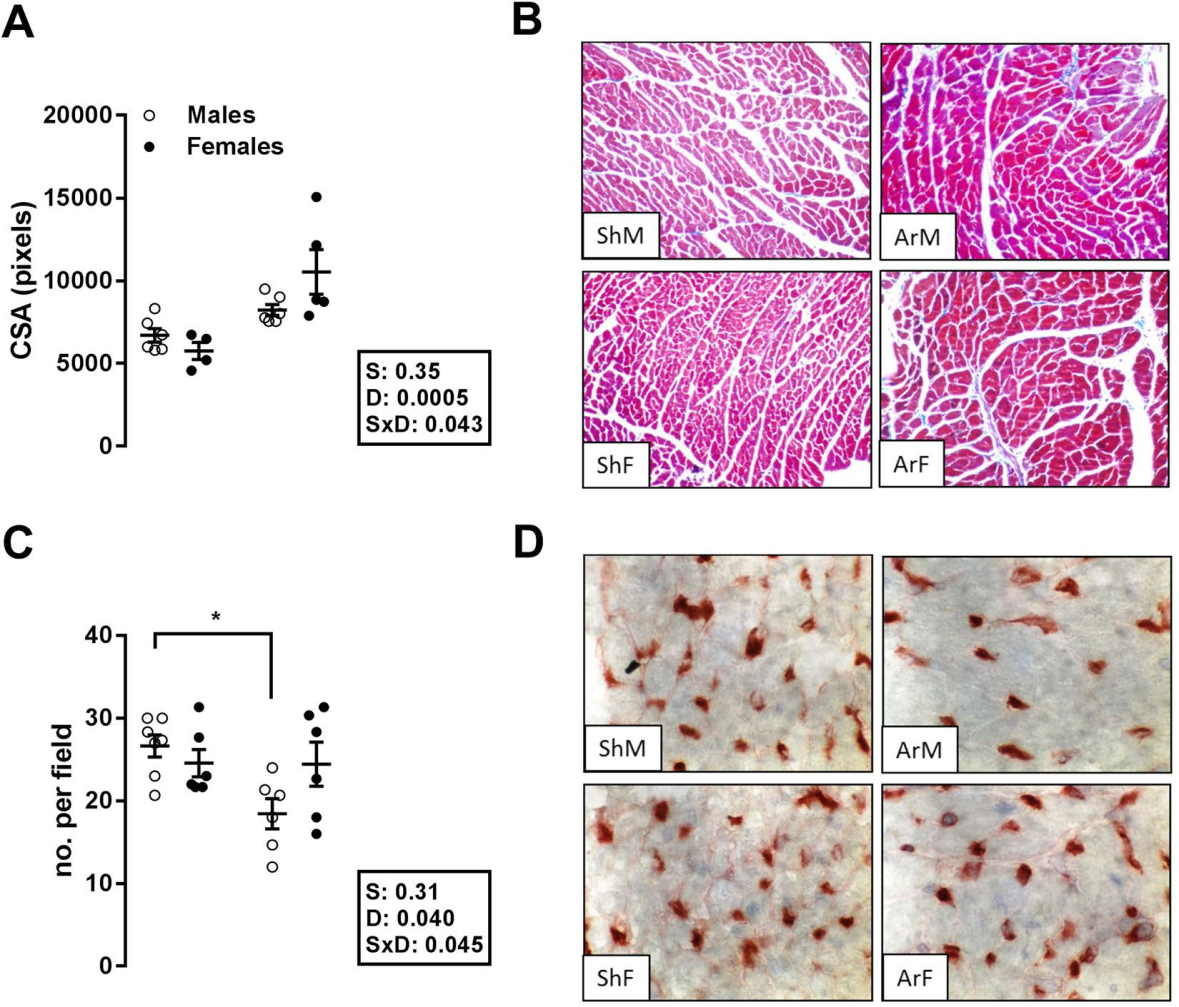
Impact of LV dilatation and hypertrophy caused by severe aortic regurgitation on cardiomyocyte cross-sectional area (Top panels; A and B) and on myocardial capillary density (bottom panels (C and D). Left panels (A and C): The results are expressed as the mean ± SEM (n = 6–7/gr.). Probability values are from a 2-way ANOVA and Holm-Sedak post-testing. *p < 0.05 or **p < 0.01 between indicated groups. Upper right panels: Representative Masson’s trichrome staining of mid-chamber short-axis LV sections (magnification x200); bottom right panels: LV sections stained with isolectin B4 coupled with horseradish peroxidase (magnification x400).

We did not observe any significant differences between groups after 6 months in terms of interstitial LV fibrosis as estimated by Masson’s trichrome staining (not shown). Cardiomyocyte cross-sectional area (CSA) was increased in AR animals compared to sham animals, as illustrated in Fig. 2A and C. LV capillary density was significantly reduced in the ArM group but remained normal in ArF animals (Fig. 2B and D).

### Markers of hypertrophy and extracellular matrix remodelling

**Figure 3:**
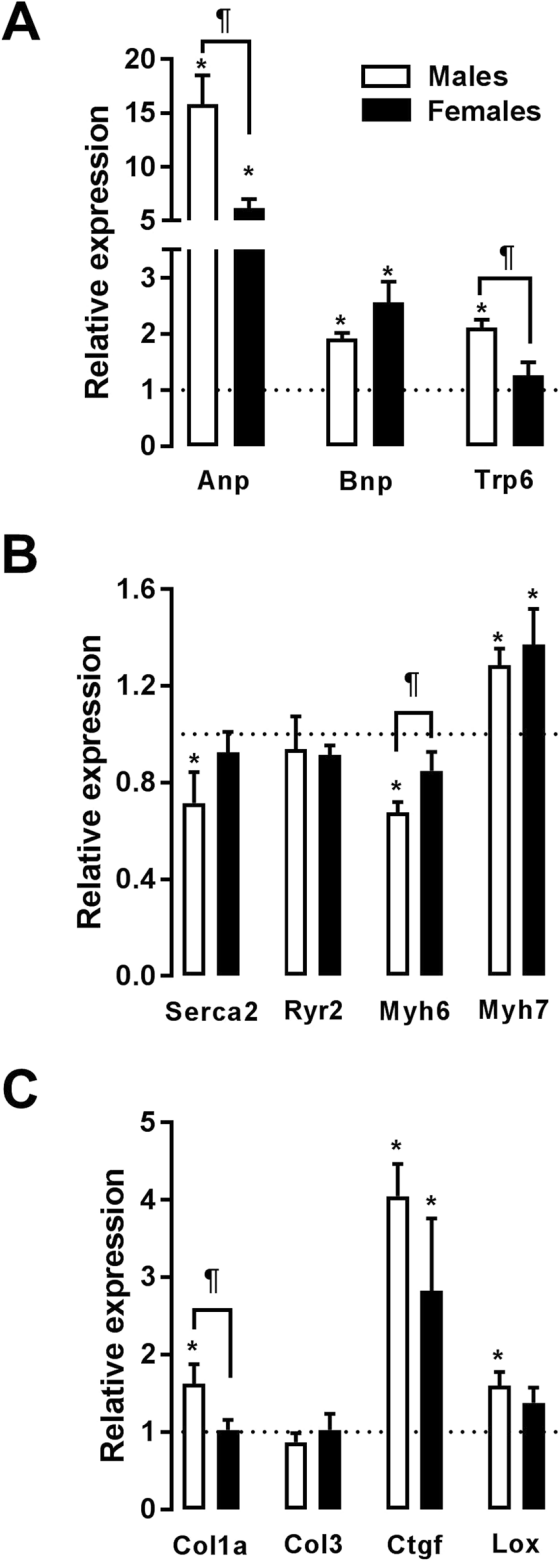
Evaluation by real-time quantitative RT-PCR of the LV mRNA levels of various hypertrophy (top two graphs; **A** and **B**) and extracellular matrix remodelling (bottom; **C**) markers. The results are reported in arbitrary units (AU) as the mean ± SEM (n = 5–6/gr.). The mRNA levels of the sham (sham-operated animals) groups (ShM and ShF) were normalized to 1 and are represented by the dotted line. White columns represent the ArM group, and black columns represent the ArF group. *p < 0.05 vs. the corresponding sham group and ^¶^p < 0.05 between the ArM and ArF groups.

As expected, Anp and Bnp gene expression was elevated in AR animals (Fig. 3A; see Table S1 for complete gene names). The increase in Anp expression was stronger in males. Trp6 (transient receptor potential channel 6) gene expression was elevated in male AR animals but not in females. The same was observed for pro-collagen 1 (Col1a). Serca2 and Myh6 mRNA levels were down-regulated only in the ArM group (Fig. 3B). Baseline levels of expression for the genes illustrated in Fig. 3 were similar between male and female sham-operated rats, with the exception of higher expression of Myh6 in females and Col3 and Lox in males (Figure S1).

### LV energy metabolism and markers

**Figure 4:**
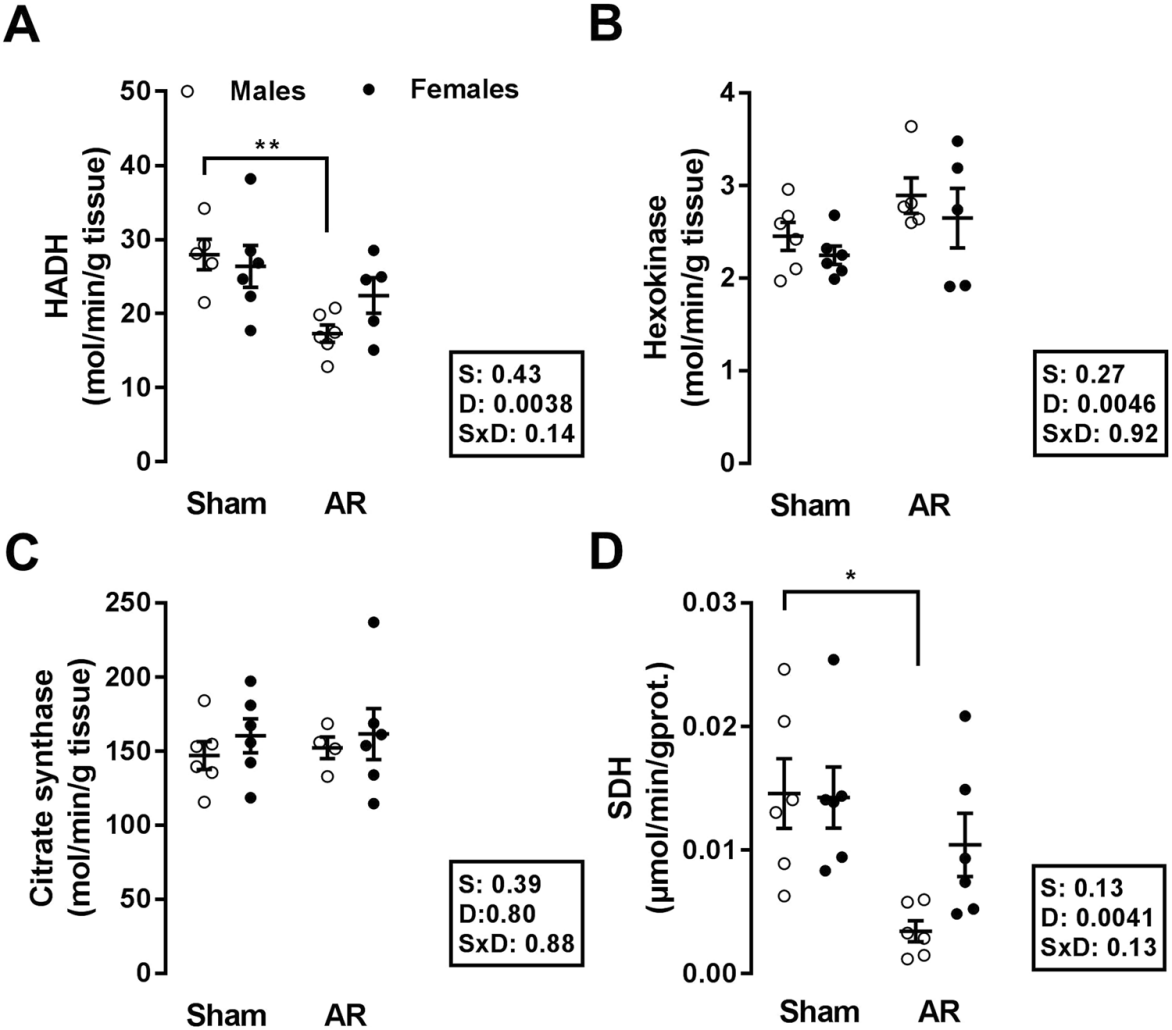
LV myocardial activity levels of enzymes implicated in fatty acid β-oxidation, glucose metabolism and mitochondrial energy production in male and female AR rats after 6 months. Hydroxyacyl-Coenzyme A dehydrogenase (HADH) (**A**), hexokinase (HK) (**B**), citrate synthase (**C**), and succinate dehydrogenase (**D**) enzymatic activities were measured in LV homogenates from 5–6 animals in each group as described in the Methods. The results are reported as the mean ± SEM. Probability values are from a 2-way ANOVA and Holm-Sedak post-testing. *p < 0.05 and **p < 0.01 between indicated groups.

Fatty acid oxidation (FAO) capacity was impaired in the LV of AR rats, as illustrated by the decreased hydroxyacyl-Coenzyme A dehydrogenase (HADH) enzymatic activity (Fig. 4A). This was statistically significant only for males, whereas it remained stable in females. Hexokinase activity was increased in both AR groups, while citrate synthase activity remained stable (Fig. 4B and C). The activity of succinate dehydrogenase, the only enzyme that participates in both the citric acid cycle and in the respiratory chain, was significantly reduced in males AR rats but not in females.

**Figure 5:**
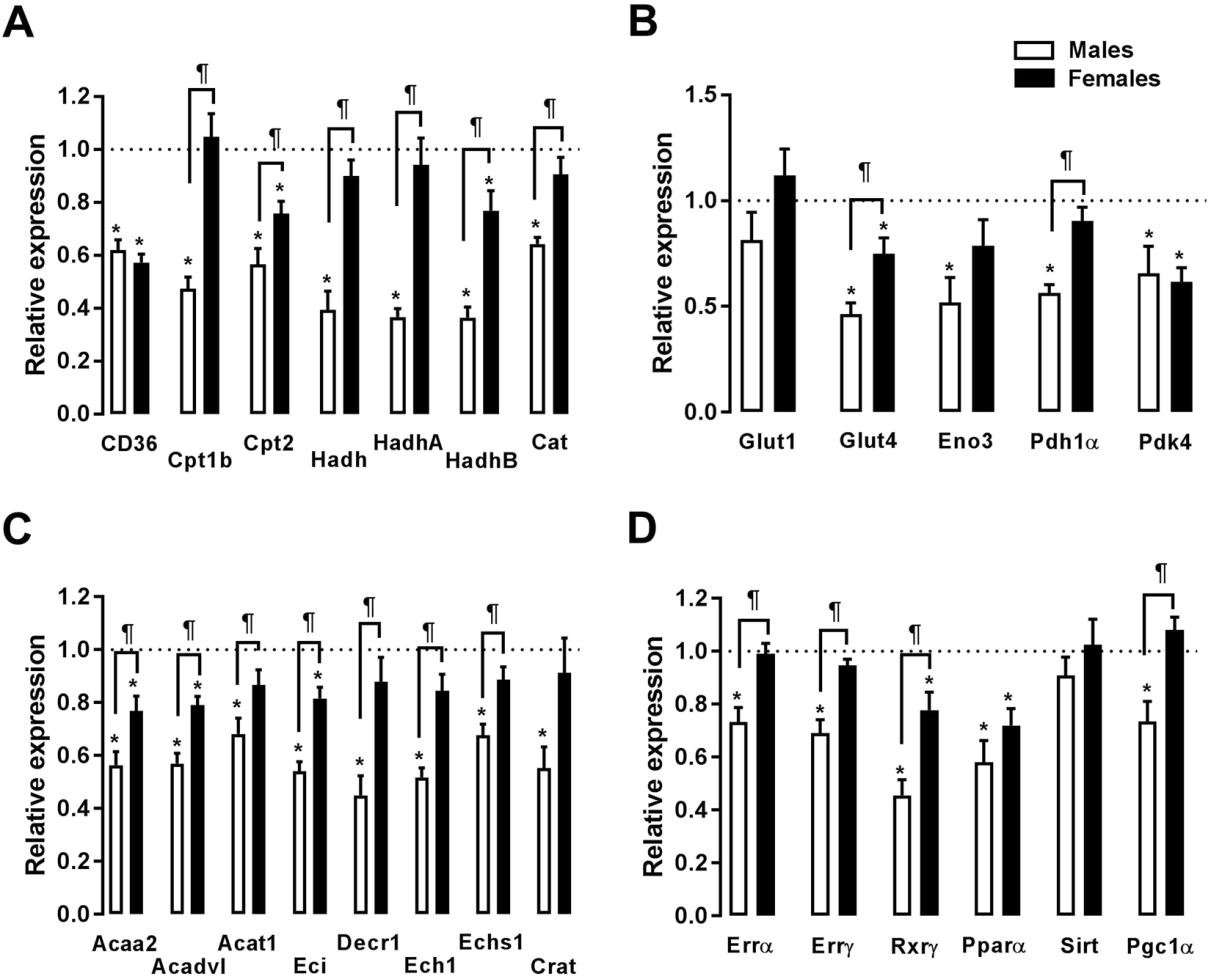
Evaluation by real-time quantitative RT-PCR of the LV mRNA levels of genes encoding for enzymes implicated in fatty acid oxidation (left panels; A and C) and glycolysis (top right panel; B) and for transcription factors related to bioenergetics control (right bottom panel; D). The results are reported in arbitrary units (AU) as the mean ± SEM (n = 5–6/gr.). The mRNA levels of the sham (sham-operated animals) groups (ShM and ShF) were normalized to 1 and are represented by the dotted line. White columns represent the ArM group, and black columns represent the ArF group. *p < 0.05 vs. the corresponding sham group and ^¶^p < 0.05 between the ArM and ArF groups.

In a previous gene expression profiling study, we observed that a number of genes related to FAO were down-regulated in AR male rats after 9 months of severe volume overload^16^. The expression levels of most of these genes were significantly decreased in male AR rats, whereas this decrease was only minimal in females (Fig. 5A and C). A similar pattern was observed for genes involved in myocardial glucose utilization (Fig. 5B). The expression of several transcription factor genes implicated in myocardial bioenergetics followed this trend, being more altered in males than in females (Fig. 5D). The baseline levels of expression of several FAO (Cpt1b, Hadh, HadhA and HadhB) and glycolysis (Glut4 and Pdh1α) genes were lower in females, as illustrated in Figure S2. On the other hand, the mRNA levels of Errγ, Sirt and Pgc1α were higher in ShF than in ShM animals.

### Markers of mitochondrial function

**Figure 6:**
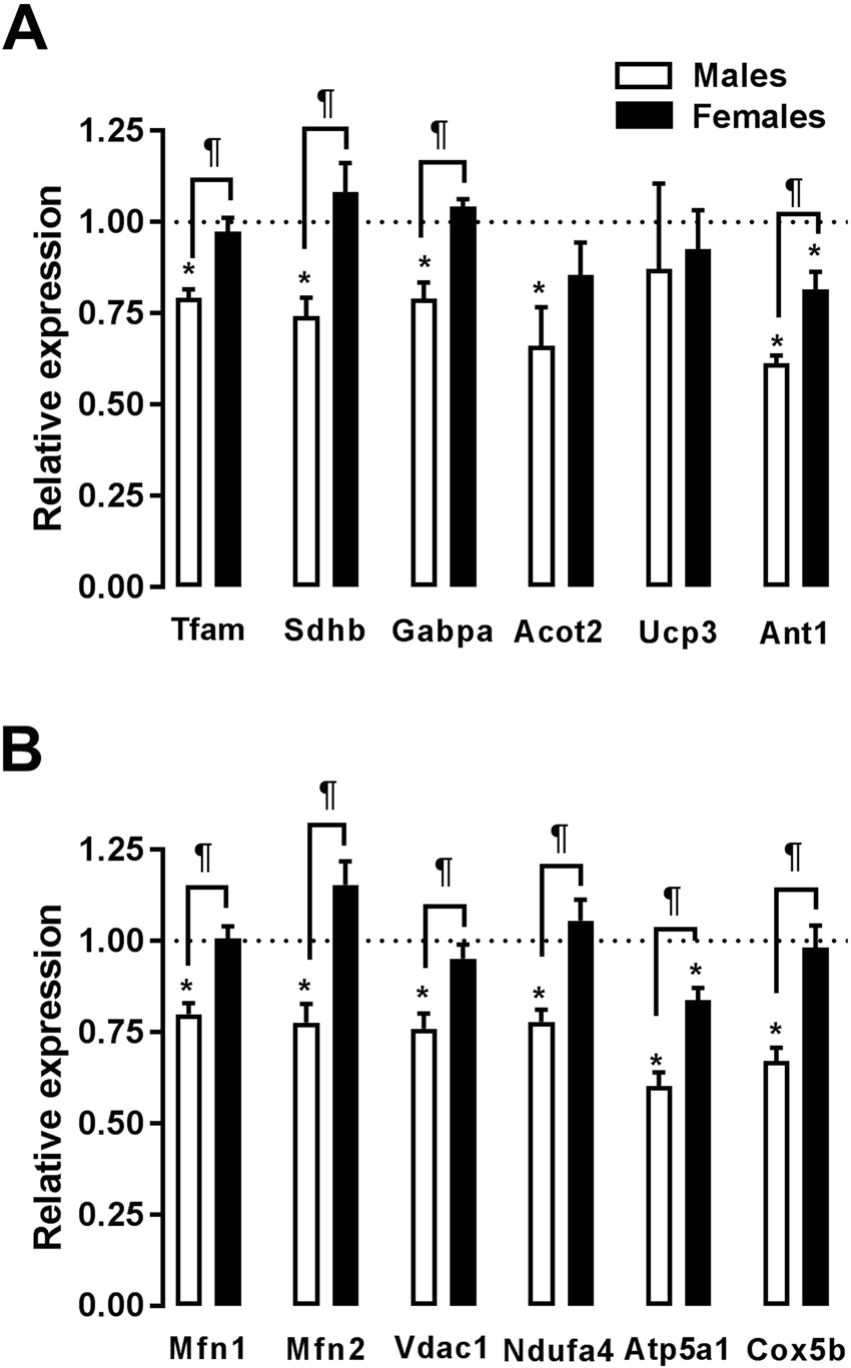
Evaluation by real-time quantitative RT-PCR of the LV mRNA levels of genes encoding for markers of mitochondrial biogenesis and function (**A** and **B**). The results are reported in arbitrary units (AU) as the mean ± SEM (n = 5–6/gr.). The mRNA levels of the sham (sham-operated animals) groups (ShM and ShF) were normalized to 1 and are represented by the dotted line. White columns represent the ArM group, and black columns represent the ArF group. *p < 0.05 vs. the corresponding sham group and ^¶^p < 0.05 between the ArM and ArF groups.

We previously identified a group of 54 genes that were associated with mitochondrial function and down-regulated in male AR rats^16^. Here, we studied a subset (12) of these genes, as illustrated in Fig. 6. The mRNA levels of genes related to the citric acid cycle, the respiratory chain or mitochondrial biogenesis were more altered in male AR rats compared to females. Baseline levels in sham-operated animals were similar between male and female animals, the only exception being Atp5a1 (Figure S3).

## Discussion

The main observation of this study is that the female sex is associated with a myocardial transcriptional profile that is closer to normal despite developing more hypertrophy in response to volume overload. There was more LV wall thickening in female AR animals, whereas chamber dilation was relatively similar, resulting in less eccentric LV remodelling compared to males. This remodelling was accompanied by lower levels of wall stress in females despite higher levels of hypertrophy and a similar degree of aortic valve regurgitation.

Most bioenergetics and mitochondrial function markers we measured remained in the normal or near-normal range in female AR animals, whereas they were clearly lowered in males. This suggests that the cardiac hypertrophy level cannot be considered to have a similar predictive value for the progression towards HF for both sexes. Relatively normal myocardial bioenergetics in females in spite of the presence of more cardiac hypertrophy may explain why their progression towards HF is slower than in males with VO^11, 13, 17, 18^. The different LV remodelling pattern (less eccentric in females) also seems to result in lower wall stress, which may play a role in maintaining normal myocardial bioenergetics. On the other hand, the increase in LV wall stress caused by VO in female rats remained important. However, the moderate transcriptional and metabolic responses of the myocardium to this significant stress are surprising.

Chronic and pathologic cardiac overload leads to neurohormonal activation. We previously demonstrated that blocking these systems (adrenergic and/or renin-angiotensin-aldosterone) led to decreased LV hypertrophy in the AR rat model and better survival^19–21^. Dent and collaborators demonstrated the presence of sex differences in neurohormonal activation in the arteriovenous fistula (ARF) VO model. After 16 weeks of VO from ARF, they observed that the protein levels of both the β1 and β2-adrenergic receptors were decreased in male rats but increased in females. Catecholamine plasma levels were also significantly lower in females. This was accompanied with increased adenylate cyclase activity, suggesting that β-AR levels and function remained mostly normal in female ARF animals^9^. This could likely contribute to the maintenance of relatively normal energy metabolism and mitochondrial function as we observed in our study, considering the importance of this system in regulating metabolism^22, 23^. We did not observe marked sex differences in the expression of the adrenergic receptors at the gene level (Figure S4) between our groups, but this does not exclude differences at another level. We previously reported increased catecholamine levels in male AR rats^24^ but only minimal changes in the mRNA levels of β-ARs^20, 24^.

Sex differences in the development of cardiac hypertrophy have been studied in several animal models. In Wistar rats with ascending aortic banding, LV hypertrophy was shown to be similar between the sexes, but progression towards HF was faster in males^25, 26^. In that PO model, the mRNA levels of Anp, Myh7 and Serca2a were all more altered in males than in females. In mice with TAC, males also developed similar levels of hypertrophy compared to females but displayed more myocardial fibrosis and progressed faster to HF^7, 27^. Interestingly, many genes associated with mitochondrial function were more down-regulated in males than in females^27^. In our study, we observed after 6 months a general reduction in the expression of many mitochondrial function markers in males, while these markers remained mostly unchanged in females. We showed in a previous study that the amount of LV mitochondrial DNA in our model after 9 months was similar to controls^16^. However, this result did not prove that mitochondrial morphology was normal in our rats. Although we did not thoroughly evaluate mitochondrial function in this study, our transcriptional profile indicates that mitochondrial function is worse in male AR rats. Moreover, we observed that the activity of succinate dehydrogenase, an enzyme implicated in both the citric acid cycle and the respiratory chain, was reduced again only in male AR rats^28^. Pgc1α, an important factor in the control of mitochondrial biogenesis and fatty acid oxidation (FAO), followed the same expression pattern^27^. Pgc1α was previously demonstrated to be positively modulated by oestrogens, which could explain our observation. In a mouse model of doxorubicin-mediated cardiotoxicity, similar sex differences were observed. The male myocardial transcription profile of genes associated with mitochondrial function was more altered than the female profile. This correlated with poorer LV systolic function and survival^29^.

We observed that FAO was impaired in male AR animals. This is in accordance with previous observations made in this model^16, 19, 30^. We also know from previous µPET studies performed in male rats with chronic AR that glucose utilization is also increased at this stage of the disease^19^. Our current results suggest that this energy substrate shift to glucose may have not taken place in female AR rats. Eccentric cardiac hypertrophy does not seem to trigger changes in myocardial substrate preference in female rats. It is interesting to note that myocardial capillary density seemed less affected in female AR rats, suggesting a better myocardial angiogenic response to severe VO^31^. This may result in not only better nutrient availability to the cardiac cells but also better oxygen availability, facilitating FAO, a highly oxygen-consuming process compared to glycolysis^32^. These observations support the need to investigate further and to study myocardial energy substrate avidity by µPET in animals of both sexes^16, 19, 33^.

In addition to Pgc1α, the expression of both oestrogen receptor-related (Err) transcription factors (α and γ) was reduced in AR males but remained unchanged in females. Err transcription factors regulate genes involved in FAO, mitochondrial biogenesis and oxidative phosphorylation^34, 35^. Their impact is probably even more important than that of Pgc1α, which is not down-regulated in patients with HF^36^. Errα is also a known downstream target of the oestrogen receptor α, which could explain the maintained mRNA levels of various genes in females^37^.

The role of oestrogens in the rat AR model of LV remodelling is still unclear. Ovariectomy does not influence the level of LV hypertrophy in the rat AR model^14^. This previous study was too small to address whether oestrogen removal influences survival. The current results suggest that the female sex provides benefits, but we are not able to determine whether oestrogens are responsible for these benefits during the evolution of eccentric LVH. Dent and collaborators reported in the ARF model that ovariectomy was associated with less hypertrophy but more LV dilation and that exogenous oestradiol administration reversed this effect^11^. Interestingly, the presence or absence of oestrogens had important effects on the myocardial β-AR content, plasma catecholamine levels and adenylate cyclase activity in ARF rats. Oestrogens can also stimulate calcineurin degradation downstream of these signalling pathways, attenuating the evolution of cardiac hypertrophy towards HF^38^. In this study, oestradiol replacement therapy in ovariectomized TAC mice was also shown to limit cardiomyocyte elongation without affecting width, suggesting a capacity of oestrogens to delay eccentric hypertrophy, decompensation and possibly HF.

### Study limitations

Our results support that the female sex provides benefits in keeping closer to normal myocardial function and bioenergetics in presence of severe VO and this despite important LV remodelling. This is in line with other observations relating to cardiac hypertrophy made in women^1, 8^. Heart adaptations to VO in women (with the exception of pregnancy) have received very little attention so far. Observations made in animals cannot necessarily be transposed to humans, and caution must be used. This study did not take into account ageing and menopause, factors that are highly relevant to heart disease in women. The effect of gonadectomy and hormone replacement therapy was not assessed in this study. We did not perform a thorough evaluation of mitochondrial function or neurohormonal status, but limited ourselves to primarily studying gene expression. It is likely that an analysis performed at the level of proteins, enzymatic activity or function would have provided more definitive conclusions.

## Conclusion

In this study, we showed that the abnormal transcriptional pattern usually associated with pathological cardiac hypertrophy does not occur in female rats with severe aortic valve regurgitation, despite an important gain in heart mass. We observed that cardiac remodelling in AR female rats leads to moderately less LV eccentricity and wall stress, better capillary density and more stable expression of many myocardial bioenergetics and mitochondrial function markers. The precise role of oestrogens during LV eccentric hypertrophy development will need to be addressed in upcoming studies.

## Methods

### Animals

Wistar male (350–375 g) and female (225–250 g) rats purchased from Charles River (Saint-Constant QC, Canada) were studied for 180 days (26 weeks). The animals were divided into four groups: male sham-operated animals (ShM; n = 11), female sham (ShF; n = 8), males with aortic regurgitation (ArM; n = 10) and female AR (ArF; n = 7). The protocol was approved by the Université Laval’s Animal Protection Committee and followed the recommendations of the Canadian Council on Laboratory Animal Care. Severe AR was induced by retrograde puncture of the aortic valve leaflets under echocardiographic guidance as previously described^39, 40^. Only animals with >60% regurgitation were included in the study. The regurgitant fraction was estimated by the ratio of the forward systolic flow time–velocity integral (VTI) to the reversed diastolic flow VTI measured by pulsed Doppler in the thoracic descending aorta.

Left ventricular and arterial pressures and dP/dT (positive and negative) were measured invasively using a dedicated catheter under isoflurane anaesthesia (5 animals/gr.)^41^. A complete echo exam was performed before AR induction and at the end of the protocol as previously described^19, 39, 42, 43^. LV mass was calculated by the following formula: 1.04((EDD + PW + SW)3 − EDD3). EDD, PW, and SW are end-diastolic diameter, posterior wall thickness, and septal wall thickness, respectively. Left ventricular mean wall stress was estimated using the formula developed by Quinones *et al*. SAP x ((EDD + ESD)/2 × (PWd + PWs)). SAP, ESD, PWd, and PWs are systolic arterial pressure, end-systolic diameter, diastolic posterior wall thickness, and systolic posterior wall thickness, respectively^15^. The hearts were harvested as previously described^14, 24^.

### Cardiomyocyte cross-sectional area

Sections from paraffin-embedded mid-LV portions were stained using Masson’s trichrome staining. Three sections per slide were studied for the evaluation of cross-sectional area (CSA) of the cardiomyocytes as described previously^44^.

### Staining for capillary density measurement

Sections of 8-µm thickness were cut from the frozen LV and were stained with isolectin B4 from *Bandeiraea simplicifolia* coupled with horseradish peroxidase (Sigma, Mississauga, ON, Canada). Capillary density was analysed in the subendocardial region of the LV myocardium (inner third) as described elsewhere^31^.

### Analysis of mRNA accumulation by quantitative RT-PCR

The analysis of LV mRNA levels by quantitative RT-PCR has been described in detail elsewhere^45^. QuantiTect (Qiagen) and IDT (Coralville, Iowa) Primer Assays (preoptimized specific primer pairs; Table S1) and QuantiFast SYBR Green PCR kits (Qiagen) were used. We also used two pairs of non-pre-optimized primers for Echs1 (5′-GCTTTCAGGGTGTCTTGATTTG-3′ and 5′-GAGCTATGCACTGCAGATAGT-3′; 95 bp transcript) and Sirt1 (5′-GAACCTCTGCCTCTACATT-3′ and 5′-CATACTCGCCACCTAACCTATG-3′; 94 bp transcript). Cyclophilin A (Ppia) was used as the control “housekeeping” gene.

### Enzymatic activity determinations

Left ventricle samples were stored at −80 °C until assayed for maximal (*V*_max_) enzyme activities. Small pieces of LV (20–30 mg) were homogenized in a glass-glass homogenizer with either 9 or 39 volumes of ice-cold extracting medium pH 7.4 (250 mM sucrose, 10 mM Tris-HCl, 1 mM EGTA) depending on the enzyme activity assayed. Enzymatic activities for hydroxyacyl-Coenzyme A dehydrogenase (HADH), hexokinase (HK), citrate synthase (CS) and succinate dehydrogenase (SDH) were determined as previously described^28, 46^.

### Statistical analysis

Results are presented as the mean ± SEM. Intergroup comparisons were performed using a 2-way ANOVA followed by a Holm-Sidak post-test. Student’s t test was used when two groups were compared directly. Statistical significance was set at a p value < 0.05. The statistical analyses were performed using Graph Pad Prism version 7.02 for Windows, Graph Pad Software (San Diego CA). All methods were carried out in accordance with the relevant guidelines and regulations.

## Electronic supplementary material

Supplementary table and figures
