# Supplementary material for: Female rats with severe left ventricle volume overload exhibit more cardiac hypertrophy but fewer myocardial transcriptional changes than males
Source: Sci Rep. 2017 Apr 7;7:729. doi: 10.1038/s41598-017-00855-9 (PMC5429715; doi:10.1038/s41598-017-00855-9)
Supplement: Supplementary file 1 — Supplementary table and figures [file 41598_2017_855_MOESM1_ESM.pdf]

Female rats with severe left ventricle volume overload exhibit more cardiac hypertrophy but fewer myocardial transcriptional changes than males.

Catherine Beaumont[1], Élisabeth Walsh-Wilkinson[1], Marie-Claude Drolet, Élise Roussel, Marie Arsenault* and Jacques Couet*.

Groupe de recherche sur les valvulopathies, Centre de Recherche, Institut universitaire de cardiologie et de pneumologie de Québec, Université Laval, Quebec City, Canada.

Running title: Sex differences in rats with aortic valve regurgitation.

[1]: These authors have contributed equally to this work.

*Corresponding authors: Jacques Couet PhD and Marie Arsenault MD

Groupe de Recherche en Valvulopathies, Centre de Recherche,

Institut universitaire de cardiologie et de pneumologie de Québec

2725, Chemin Sainte-Foy, Québec, (Québec), Canada, G1V 4G5

Phone: 1-418-656-4760; Fax: 1-418-656-4509

Email: jacques.couet@med.ulaval.ca or marie.arsenault@criucpq.ulaval.ca

Table S1. Primer Assays used in qPCR analysis of gene expression.

mRNA	Symbol	Cat. No.	Amplicon (bp)
acetyl CoA acyltransferase 2	Acaa2	Rn.PT.58.5300756	111
acyl CoA déshydrogenase, very long chain	Acadvl	Rn.PT.58.13279450	147
acetyl CoA acetyltransferase 1	Acat1	Rn.PT.58.18447027	102
acyl-CoA thioesterase 2 (Acotinase-2)	Acot2	QT00407204	77
solute carrier family 25, member 4	Ant1, Slc25a4	QT01081633	143
ATPase, Ca++ transporting, cardiac	Atp2a2, Serca2a	QT01081500	96
ATP synthase, H+ transporting, mitochondrial F1 complex, alpha subunit 1, cardiac muscle	Atp5a1	Rn.PT.58.6992257	100
solute carrier family 25 (carnitine/acylcarnitine translocase), member 20	Cat, Slc25a20	Rn.PT.58.6247859	116
procollagen-1 alpha-1	Col1a	Rn.PT.58.7562513	134
procollagen-3 alpha-1	Col3	QT01083537	111
cytochrome c oxidase subunit 5B	Cox5b	Rn.PT.58.8467954	138
cartinine palmitoyltransferase 1b, muscle	CPT1b	QT01084069	98
carnitine palmitoyltransferase 2	CPT2	QT00186473	150
carnitine O-acetyltransferase	Crat	Rn.PT.58.36282119	97
connective tissue growth factor	Ctgf	QT00182021	102
2,4-dienoyl CoA reductase 1	Decr1	Rn.PT.58.44352482	120
enoyl CoA hydratase 1	Ech1	Rn.PT.58.33832465	99
enoyl CoA delta isomerase 1	Eci1	Rn.PT.58.37662439	119
enoyl CoA hydratase, short chain, 1, mitochondrial	EchS1	Non-optimized primers	95
enolase 3, beta	Eno3	QT00180138	106
estrogen related receptor, alpha	Erra	Rn.PT.58.5170310	111
estrogen related receptor, gamma	Errg	Rn.PT.58.8028733	109
fatty acid translocase/CD36, similar to	FAT/CD36	QT01702680	81
GA binding protein transcription factor, alpha	Gabpa	Rn.PT.58.12555216	137
solute carrier family 2 member 1	Glut1, Slc2a1	QT00178024	85
solute carrier family 2 member 4	Glut4, Slc2a4	QT00175931	146
hydroxyacyl-CoA dehydrogenase	Hadh	Rn.PT.58.17867024	135
hydroxyacyl-CoA dehydrogenase alpha	Hadha	Rn.PT.58.46222281	138
hydroxyacyl-CoA dehydrogenase beta	Hadhb	Rn.PT.58.7613498	130
Lysyl oxidase	Lox	QT00185591	148
mitofusin 1	Mfn1	Rn.PT.58.44207597	106
mitofusin 2	Mfn2	Rn.PT.58.13375660	124

myosin, heavy polypeptide 6, cardiac	Myh6	QT00190267	127
myosin, heavy polypeptide 7, cardiac	Myh7	QT00189504	144
NADH:ubiquinone oxidoreductase subunit A4	Ndufa4	Rn.PT.58.11318539	134
natriuretic peptide precursor type A	Nppa, Anp	QT00366170	94
Natriuretic peptide precursor type B	Nppb	Rn.PT.58.5595685.g	108
pyruvate dehydrogenase alpha 1	pdha1	QT01830220	93
pyruvate dehydrogenase kinase, isozyme 4	Pdk4	QT00189287	145
peroxisome proliferator activated receptor gamma, coactivator 1 alpha	Pgc1α	QT00189196	108
peroxisome proliferator activated receptor alpha	Pparα	QT00176575	66
cyclophilin a	Ppia	QT00177394	106
retinoid X receptor gamma	Rxrγ	Rn.PT.58.6519292	103
ryanodine Receptor 2, cardiac	Ryr2	QT01811936	60
succinate dehydrogenase complex iron sulfur subunit B	Sdhb	Rn.PT.58.9629097	110
sirtuin 1	Sirt1	Non-optimized primers	94
transcription factor A, mitochondrial	Tfam	Rn.PT.58.13772978	124
transient receptor potential cation channel, subfamily C, member 6	Trpc6	QT00195804	101
uncoupling protein 3	Ucp3	QT00176589	103
voltage-dependent anion channel 1	Vdac1	Rn.PT.58.37188705	116

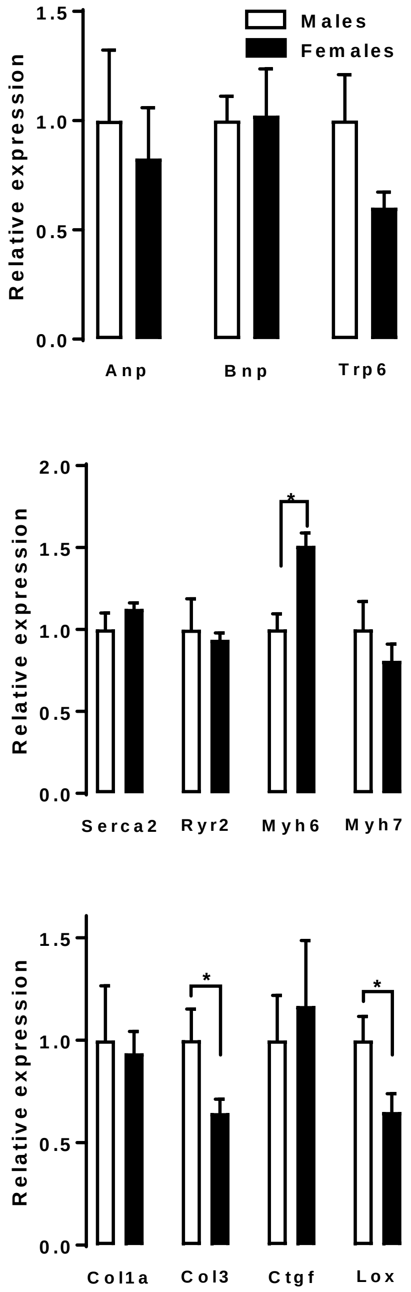

Figure S1: Evaluation by real-time quantitative RT-PCR of the LV mRNA levels of various hypertrophy (top two graphs) and extracellular matrix remodeling (bottom) markers in male (white) and female (black) sham-operated animals. Results are reported in arbitrary units (AU) as mean ± SEM (n=5-6/gr.). ShM mRNA levels were normalized to 1. *: $p<0.05$ between groups.

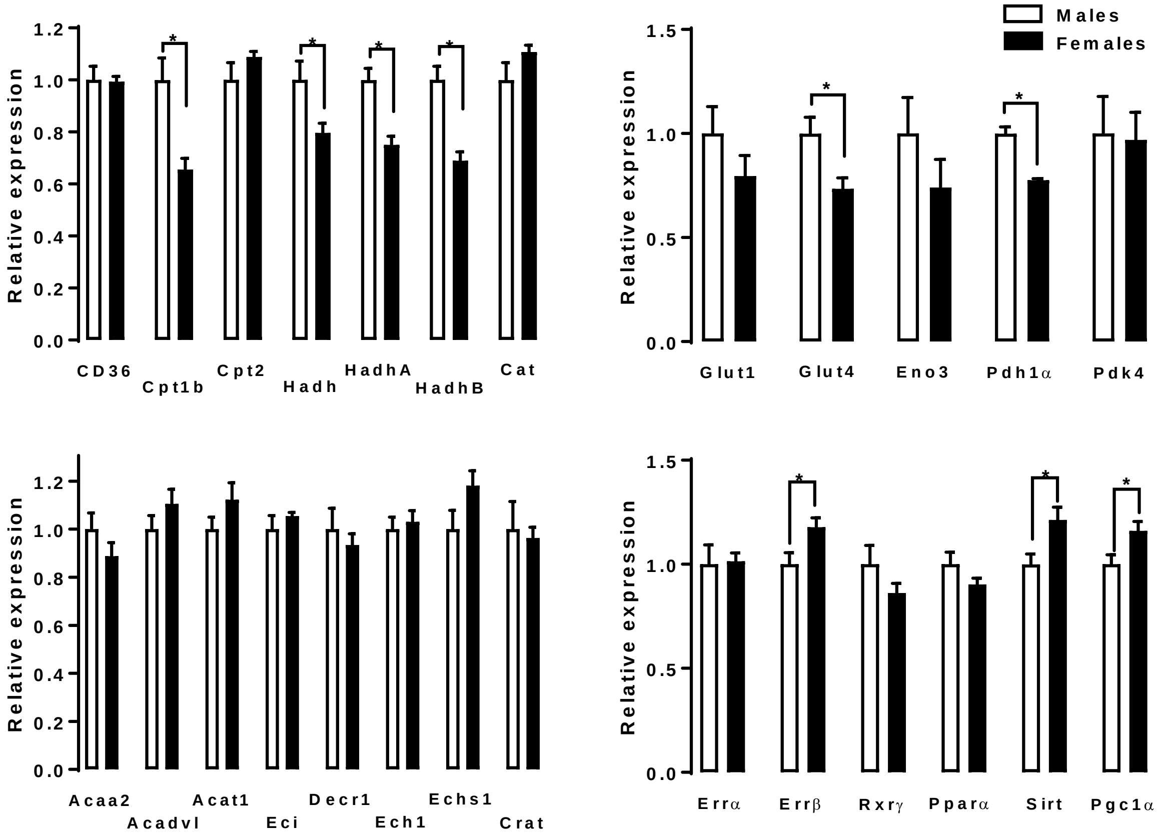

Figure S2: Evaluation by real-time quantitative RT-PCR of the LV mRNA levels of genes encoding for enzymes implicated in fatty acid oxidation (left panels), glycolysis (top right panel) and or for transcription factors related to bioenergetics control (right bottom panel) in male (white) and female (black) sham-operated animals. Results are reported in arbitrary units (AU) as mean ± SEM (n=5-6/gr.). ShM mRNA levels were normalized to 1. *: $p<0.05$ between groups.

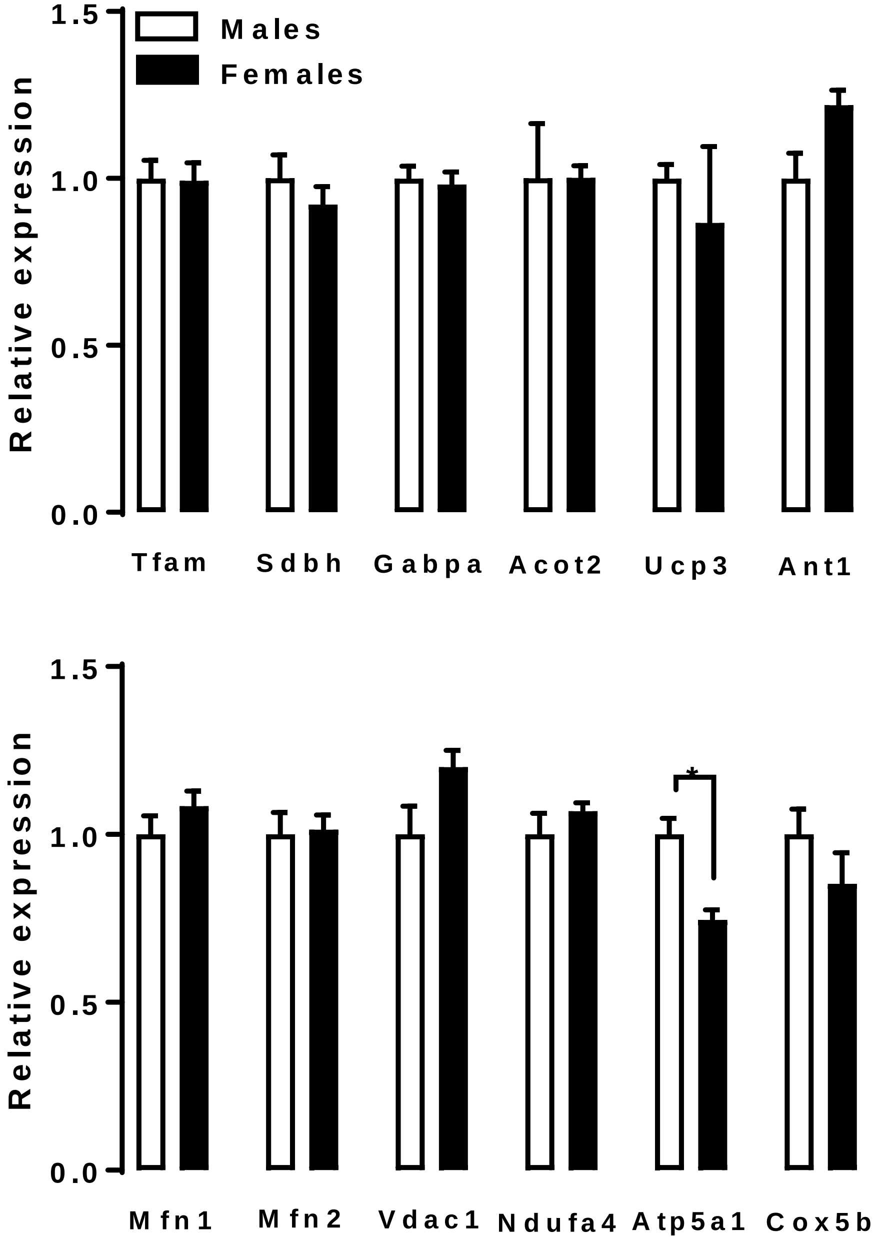

Figure S3: Evaluation by real-time quantitative RT-PCR of the LV mRNA levels of genes encoding for markers of mitochondrial biogenesis and function in male (white) and female (black) sham-operated animals. Results are reported in arbitrary units (AU) as mean ± SEM (n=5-6/gr.). ShM mRNA levels were normalized to 1. *: $p<0.05$ between groups.

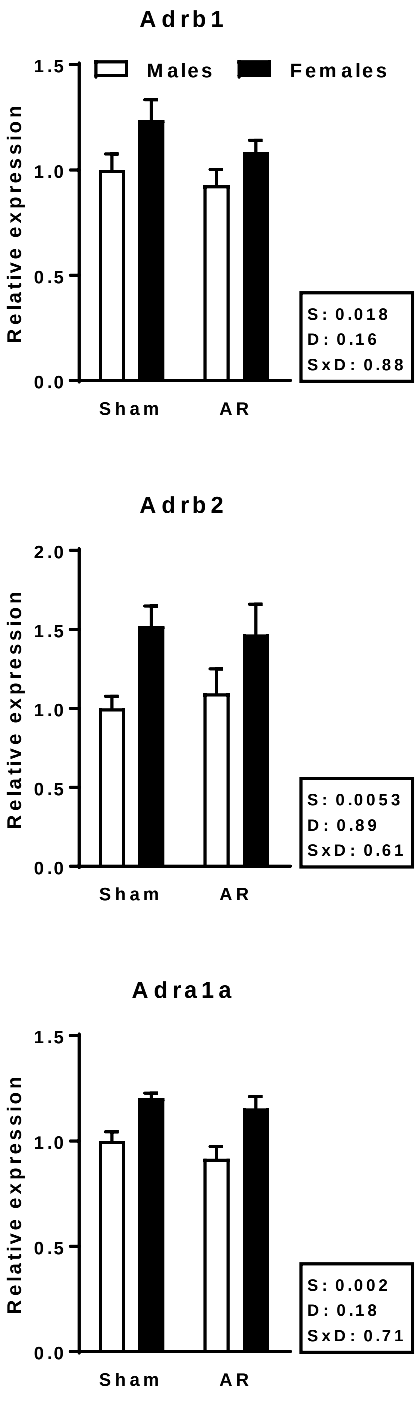

Figure S4: Evaluation by real-time quantitative RT-PCR of the LV mRNA levels of genes encoding for adrenergic receptors (beta1, beta2 and alpha1 in male (white) and female (black) sham-operated animals. Results are reported in arbitrary units (AU) as mean ± SEM (n=6/gr.). ShM mRNA levels were normalized to 1.
